# Editorial: Community series in resident memory T cells: guardians of the balance of local immunity and pathology, volume II

**DOI:** 10.3389/fimmu.2024.1401196

**Published:** 2024-06-10

**Authors:** Nicholas P. Goplen, Chaofan Li, Shiki Takamura

**Affiliations:** ^1^ Department of Physiology and Biomedical Engineering, Mayo Clinic, Rochester, MN, United States; ^2^ Division of Infectious Disease and International Health, Department of Medicine, University of Virginia, Charlottesville, VA, United States; ^3^ Department of Immunology, Faculty of Medicine, Kindai University, Osaka, Japan

**Keywords:** memory T cell, cancer, infection, vaccine, immunotherapy

What happens between the time a T cell loses its naivety, expands thousands-fold, and all its clonal progeny die can impact T cell sentinel functions in tissues. Holistically understanding the dynamic control that imparts heterogeneous functional responses of tissue-resident memory T cells (T_RM_) could clinically aid in balancing health and disease at the level of local immunity. Notably, surrogate measurements of the functional quality of T_RM_ are quickly becoming a favored prognostic tool in the fight against cancer, with signs of the same on the horizon for local infections, allergies, and autoimmune diseases. If you are interested in how T cells in yourself, others, or model organisms, act, or how human T cells in mice act the way they do, we have some recommended reading for you exploring origin stories and fate decisions that influence “The Guardians of The Tissue”.

Did somebody have the gall to tell you your work needs more clinical relevance and you now find yourself looking for a road map of how to establish it, to validate your existence and appease the grant gods at the same time? Look no further, as Fujiki et al. have paved the way. This one did not even use the word *resident* once, but we could not put it down. Vitamin A metabolite signaling promotes CD4^+^ and CD8^+^ T cell effector function after productive T Cell Receptor (TCR) signaling. Fujiki et al. discovered that T cells do the metabolizing themselves. But not in a way that makes them full-fledged terminal effectors; the good stuff (retinoic acid) that encourages that commitment comes from exogenous sources such as tumor microenvironments. However, when the goal is memory generation, too much of the good stuff is a bad thing, or perhaps, just cancers’ nefarious way of making sure no memories remain. To learn more on the epigenetic modifications of this intrinsic pathway that controls T cell fate, tune in to this riveting article with classic competition experiments, tertiary memory generation, and xenografts. We recommend you get your lab some LE540 yesterday; this paper could cause a supply crunch driven by T cell fate enthusiasts!

The right and the left colon differ in terms of their embryonic origin, vascularization, innervation, and gut flora. Talhouni et al. make it obvious from the get-go that, since left- and right-sided colorectal cancers (CRC) develop in different environments, the approach to understand and treat them should not be “one side fits all”. Since high numbers of T cells inside tumors (tumor hotness) is a better prognostic indicator than the stage of the tumor itself, what if you could find out something unique about those T cells in tumors that improved our ability to monitor disease progression and dynamically guide treatment decisions? Many independent studies of cancers throughout the body have done just that. This massive undertaking, where over 900 patients with CRC contributed to findings that are going to improve clinical assessment and treatment of left- and right-sided CRC, is the largest imaging study of T_RM_ in tumor microenvironments. Machine learning trained on unique combinations of three immunofluorescent markers (CD8α, CD103, and CD39) on tissue microarray biopsies aided quantitation of cellular densities in right- and left-sided hot and cold tumors. They speculate on why their CRC findings are not “one side fits all” in the context of recent understandings of checkpoint blockade mechanisms of action and what they know of colon sidedness, with a discussion as captivating as the article was throughout.

Presumably, there is prognostic value of absolute counts of lymphocyte subsets in the circulation over measuring frequency of the same; an easy adjustment to make for flow cytometry based clinical measurements. Zhang et al. test this principle with transparent methods to predict progression-free survival and immune therapy efficacy for patients with advanced non-small cell lung cancer. They suggest studies unwittingly including T memory stem cells without separating them from their corresponding naïve pools can misguide study conclusions. These blood screening methods could guide patient specific monitoring and treatment and could find relevance in many T cell-mediated diseases.

When you hear IL-7 mentioned in everyday conversation, do you ever just think about how its receptor mediates STAT-5 dependent survival in T cells during early stages of development and naïve existence? Do you then try and recall which cytokine receptors all share the common gamma chain? Or does it hit deeper and conjure up vivid pictures of tonic TCR signaling in survival and homeostatic proliferation leading to memory differentiation in lymphopenic conditions described in landmark papers from Charlie Surh’s group circa 2000 that quickly evolved into Kaech et al. discovering IL-7Rα could selectively predict T cell fates early in a response demonstrating its functional importance for CD8^+^ T cell memory generation? This thorough and well-organized review of the role of IL-7 in T cell biology by Zhao et al. covers this and so much more. It turns out survival is somewhat key to generating long-lasting memory T cells, an indisputable goal in cancer immunotherapy regimens. Efforts to add IL-7 to immunotherapies in preclinical and clinical trials. Improvements on IL-7 adjuvant are discussed including altering formulations, chaperones, and candidate adjuvants for IL-7 adjuvants.

We are often left with the same somber impression as these authors that the only good measure of a T cell response in therapeutic development pipelines is clinical success or failure. Nerves are immediately hit, reminding us of our repressed thoughts: *vaccines are optimized for their antibody production; those that induce cellular immunity do so because of happy accidents, not design.* Imagine how much good we could do for so many more diseases if measuring resting antigen-specific memory T cells was as convenient (or more) as antibody titers from serum. The goal is simple, noble, and rightfully reverent of T cell memory, namely, to develop a measure of anti-tumor CD4 and CD8 memory T cell responses that can be “easily standardized”. Gao and Bergman apply bulk TCRseq clonal analysis in a model of curative viral onco-immunotherapy. Although clonal expansion is 10 times greater in the peritoneum upon tumor rechallenge, ~70% of the high-responding memory T cell clonotypes were also high responders in the spleen. This is encouraging, indicating the plausibility of blood monitoring. The importance of such a patient-specific method in tumor vaccine models is demonstrated as each mouse mostly had unique clones (compared within group) that were high responders to the same antigen (private TCR repertoire). While the conclusions indicate responsive anti-tumor memory T cell clones cannot currently be predicted/measured at resting state alone, the method can certainly aid discovery and hypothesis testing in preclinical models after antigen rechallenge.

Select serine-proteases from the granzyme family are traditionally recognized for their intracellular roles (*in trans*) in perforin-dependent immune-targeted cell death, a major mechanism of both innate and adaptive immunity. Non-canonical functions of these proteases include tunneling through tissues, cleavage of viral proteins that mediate infection, production of autoantigens, collagen degradation, fibroblast activation, wound repair, and biological aging. Thus, as both a family and individual molecules, granzymes are functionally diverse. Very little is known about granzyme C (human ortholog(ue), granzyme H). In a venture into the unknown, Lujan et al. looked at the usual [granzyme] suspects - innate and adaptive T cell subsets in the skin of mice at steady-state. About ¼ of dendritic epidermal [γδ] T cells expressed granzyme C, often in the dendritic extensions between keratinocytes which could be functionally suggestive. These cells acutely increased granzyme C expression in response to cutaneous poxvirus infection. 30 days later ~15% of steady-state dermal and epidermal CD8^+^ T_RM_ also expressed granzyme C. Secondary exposure to virus with or without cognate antigen, cognate antigen alone, or IL-15 alone augmented granzyme C expression to various degrees in T_RM_. We and the authors await conditional knockouts of granzyme C to assess cell-specific bystander and antigen-specific functions in the skin.

This Research Topic includes a perspective with a data-tease that supports the hypothesis that lung pathogens should enter the lung to induce a “quality” CD8^+^ T_RM_ cell response (increased quantity and diversity of functionality, in our opinion). Viruses with lung tropism might be “better” at T_RM_ differentiation in general, versus viruses with no affection for the lung that were forced to abide there through the machinations of Macedo et al. While ostensibly they anthropomorphize pathogens to help us understand the big questions we should all ponder, are the authors really just ascribing that voice to CD8 T cell antigens and local microenvironments in which memories are made? You decide. Regardless, *we and they* think this may also have implications for mRNA vaccinations, which supports their subtle message of getting quality TCR-pMHC interactions to where you want the sensitive, rapidly responding memories while also keeping a less zealous polyclonal backup pool to guard against mutating pathogens.

Zhou et al. raise the bar for establishing vaccine efficacy for communicable diseases. In short, they provide a missing proof of principle demonstrating the value of both systemic, but especially respiratory tract, immunization to the population writ large. They demonstrate substantial and stable CD8^+^ T_RM_ establishment in airways and lung parenchyma after intranasal vaccination with Sendai virus nucleoprotein. After infecting with transmittable murine parainfluenza virus (Sendai) that encodes luciferase, parainfluenza replication was well contained in vaccinated hosts - longitudinally measured with bioluminescence as a demonstrated surrogate for viral titers. Those that received intranasal vaccine allowed very little, if any, viral replication with all other groups reaching 10,000-fold initial fluorescent intensity in the first three days of infection. This sterilizing immunity was dependent on CD8 T cells, IFNγ, IFNγR, and, to a lesser extent, perforin. Cohousing of a mouse infected 150 days after intranasal vaccination produced no new infections among the naïve cohoused population compared to sham nucleoprotein and unvaccinated mice spreading infection to 100% of cohabitants. While subcutaneous vaccinations led to viral spread, the secondary contacts exhibited well-controlled infections compared to sham and unvaccinated mice. These studies suggest that vaccinations that establish T_RM_ are paramount for herd immunity to communicable respiratory viruses. Optimizing respiratory vaccines based on limiting transmission in preclinical models may substantially improve vaccine efficacy and benefit humanity.

Most adults worldwide have been infected with the herpesvirus human cytomegalovirus (CMV). In case of immune dysregulation, reactivation of latent CMV carries a severe or even fatal risk in immunocompromised individuals. This reactivation is kept in check by CD8 T cells but the location and nature of those CD8 T cells in humans is poorly understood. White adipose tissue (WAT) has been considered an endocrine organ for decades and, more recently, an immune organ that sponsors fat-associated lymphoid clusters (FALCs). FALCs are highly enriched in resident memory CD8 T cells (T_RM_) against chronic and/or latent viruses with WAT and non-WAT tropisms. Redruello-Romero et al. used high-throughput sequencing analyses of the TCR locus in subcutaneous sWAT, lesser studied omental oWAT, and liver samples from 11 obese donors. Hyperexpanded clones from the whole repertoire were particularly enriched in each of these tissues relative to blood, consistent with their findings that most were tissue-resident memory phenotypes. The oWAT contained the most cells predicted, and confirmed, to react to CMV epitopes. This work highlights omental WAT depots in humans as a site where latency of CMV may be kept in check over the lifespan by resident memory CD8 T cells that preferentially reside there.

We hope this volume leaves you in awe at the quality and ingenuity of tools being used to explore cutting edge questions and has you pondering the future of T cell responses throughout the body.

## Author contributions

NG: Conceptualization, Supervision, Writing – original draft, Writing – review & editing. CL: Supervision, Writing – review & editing. ST: Supervision, Writing – original draft, Writing – review & editing.

## In memoriam of Toshinori Nakayama

We thank all the authors, reviewers, readership, and, in particular, Dr. Toshinori Nakayama, *in memoriam*, for his contributions to humankind. We regret to announce our co-guest editor on this Research Topic passed away on November 2, 2023. It goes without saying that he made enormous contributions to the field of immunology. In his later years, he especially devoted himself to the training of the next generation of researchers. He never told anyone of his illness and continued his research until the end of his life, fulfilling the words he left his members at the Annual Meeting of the Japanese Society for Immunology in January 2024, where he was supposed to host, touching the hearts of all attendees in a video message that began: “Keep running as best you can 全力で走れ.”

